# Existential distress and social alienation in adolescents with allergic rhinitis: the serial mediating roles of psychological inflexibility and experiential avoidance

**DOI:** 10.3389/fpubh.2026.1846579

**Published:** 2026-06-23

**Authors:** Le Huang, Yi Wang, Shi Huang

**Affiliations:** Department of Otorhinolaryngology–Head and Neck Surgery, Clinical Medical College, The First Affiliated Hospital of Chengdu Medical College, Chengdu, China

**Keywords:** adolescent patients with allergic rhinitis, existential distress, experiential avoidance, psychological inflexibility, social alienation

## Abstract

The chronic symptoms of allergic rhinitis not only compromise patients’ physical health but also exert detrimental effects on sleep quality, social engagement, and emotional wellbeing, particularly among adolescents. Although previous research has established that existential distress in patients with chronic diseases adversely affects social functioning, few studies have examined the relationship between existential distress and social alienation or the underlying mechanisms in adolescent patients with allergic rhinitis. To address this gap in the existing literature, the present study recruited 425 adolescent patients with allergic rhinitis from three tertiary Grade-A hospitals in Sichuan Province, China, between December 2025 and February 2026. The survey battery comprised the Existential Distress Scale, the Psychological Inflexibility Scale, the Experiential Avoidance Scale, the Social Alienation Scale, and requisite sociodemographic items. Results indicated that existential distress was significantly and positively associated with social alienation among adolescent patients with allergic rhinitis (*r* = 0.479, *p* < 0.001). In the cross-sectional mediation model, psychological inflexibility and experiential avoidance showed a significant serial indirect association on this relationship (Effect = 0.018, SE = 0.007, 95% CI = [0.006, 0.033]). These findings not only advance the theoretical understanding of the mechanisms underlying social functioning impairment in adolescents with chronic diseases but also provide clear directional guidance for the design of clinical interventions grounded in Acceptance and Commitment Therapy.

## 1 Introduction

Allergic rhinitis is an IgE-mediated, non-infectious chronic inflammatory disease of the nasal mucosa (1), primarily characterized by recurrent episodes of nasal pruritus, sneezing, rhinorrhea, and nasal congestion (2). Epidemiological evidence indicates that the prevalence of allergic rhinitis among children and adolescents has been increasing steadily (3, 4), with reported rates among Chinese adolescents ranging from 17.9 to 24.9% (5). Notably, adolescence represents a developmental period during which self-awareness undergoes rapid differentiation, identity construction remains incomplete, and the emotion regulation system is still maturing (6). The chronic symptom burden imposed by allergic rhinitis not only impairs adolescents’ physical health (7) but also erodes their psychological wellbeing and social functioning by compromising sleep quality, restricting daily social participation, and undermining emotion regulation capacity (8). Nevertheless, prior research has predominantly focused on pharmacological treatment, disease management strategies, and the pathophysiological mechanisms of allergic rhinitis (9–11), leaving a significant gap in our understanding of the psychological responses elicited by the illness experience and the intrapsychic mechanisms through which these responses translate into social functioning impairment among adolescent patients.

Existential distress refers to the profound anxiety, helplessness, and spiritual anguish that individuals experience when confronting the fundamental questions of human existence (12). In recent years, research on existential distress has progressively expanded beyond the domain of severe somatic illnesses such as cancer into the broader field of chronic diseases (13), suggesting that even non-life-threatening chronic conditions can trigger deep existential reflection. For adolescent patients with allergic rhinitis, the recurrent nature of the disease, the unpredictability of symptoms, and the sustained interference with daily life may profoundly undermine their fundamental sense of security regarding personal health and their belief in the controllability of life (14). More importantly, adolescents who are at a critical juncture of identity formation possess rapidly developing abstract cognitive abilities that enable them to reflect on deeper questions concerning the meaning of existence, personal worth, and the fairness of life (15, 16). However, the prolonged symptom burden and functional limitations associated with allergic rhinitis may transform such reflections into persistent existential questioning. For instance, whether a repeatedly constrained life retains any value or meaning. These existential distress experiences are not merely episodes of low mood or generalized anxiety (17); rather, they involve fundamental questioning of the very nature of one’s existence and thus possess a deeper psychological pervasiveness.

Social alienation denotes the subjective experience of psychological isolation and relational disconnection from others, groups, or broader social systems during the course of social interaction (18, 19). Peer relationships constitute a central driving force in the socialization process of adolescents (20), and the fulfillment of social belonging needs is closely associated with self-esteem construction, emotional stability, and overall psychological wellbeing (21). Prior research has documented that adolescents with chronic diseases experience elevated levels of social alienation (22), primarily attributable to disease-imposed restrictions on social participation, peer stigmatization arising from symptom visibility, and self-referent social comparisons triggered by illness identity (23, 24). When these disease-related barriers to social engagement accumulate over time, they may become progressively internalized as pervasive social alienation, manifesting as subjective perceptions of exclusion from the peer group, an inability to integrate into normative social life, and a sense that one’s existence lacks social significance (25). Consequently, elucidating the psychological mechanisms underlying social alienation among adolescents with allergic rhinitis holds substantial theoretical importance and clinical value.

Yalom (26) identified existential isolation as one of the fundamental givens of human existence, positing that when individuals become acutely aware of their fundamental aloneness, this recognition may generalize to the interpersonal domain, attenuating both the motivation and the capacity to establish meaningful connections with others. However, the relationship between existential distress and social alienation has not been empirically examined in prior research, particularly within the population of adolescents with chronic diseases. More critically, merely describing the association between existential distress and social alienation is insufficient to provide definitive theoretical guidance for clinical intervention. The more pivotal scientific question concerns the specific psychological mechanisms through which existential distress is transformed into social alienation.

According to Acceptance and Commitment Therapy theory (27), the core of psychopathology lies not in negative emotions or distressing cognitions per se, but rather in the psychological inflexibility that individuals exhibit when responding to these internal experiences. Psychological inflexibility is a comprehensive psychological process characterized by excessive dominance of internal cognitive and emotional reactions, cognitive fusion with negative thought content, attachment to a conceptualized self, and behavioral entrapment in ineffective patterns (28). When individuals exhibit high levels of psychological inflexibility, their behavior is no longer flexibly guided by personal values and contextual demands but is instead rigidly governed by internal negative thoughts and emotional reactions (29). It is noteworthy that the deep existential questioning regarding illness meaning and self-worth inherent in existential distress, when superimposed upon elevated psychological inflexibility, may engender ruminative fusion with these existential cognitions (30). Specifically, rather than accepting and accommodating existential distress as a normal psychological experience, individuals come to equate it with immutable reality, becoming cognitively fused with rigid thought content such as “my life has no meaning” or “I am fundamentally flawed.” Previous research has confirmed that psychological inflexibility is significantly associated with higher levels of psychological distress (31), lower quality of life, and poorer social functioning. However, no prior study has examined whether psychological inflexibility mediates the relationship between existential distress and social alienation.

Experiential avoidance refers to an individual’s unwillingness to remain in contact with private internal experiences, coupled with deliberate attempts to alter the form, frequency, or situational sensitivity of these experiences (32, 33). Experiential avoidance and psychological inflexibility are theoretically linked by a hierarchical causal progression. Psychological inflexibility represents a rigid dispositional stance toward internal experiences (34), whereas experiential avoidance constitutes the concrete behavioral externalization and functional consequence of psychological inflexibility (35). Specifically, individuals with elevated psychological inflexibility are more inclined to appraise internal distress as intolerable and requiring elimination, thereby adopting avoidant behavioral strategies. In the specific context of adolescent patients with allergic rhinitis, when the deep existential questioning evoked by existential distress is experienced as an unbearable psychological threat through the cognitive fusion processes of psychological inflexibility, experiential avoidance may be activated as a coping strategy. Patients may attempt to avoid disease-related distressing cognitions, evade social situations that provoke existential anxiety, and suppress internal questioning about their self-worth and the meaning of their existence. However, when individuals adopt experiential avoidance strategies, they avoid not only the painful internal experiences themselves but also the external contexts and behaviors associated with those experiences. Over time, sustained avoidance of social situations leads to the progressive weakening of social connections, the deterioration of social skills, and a further decline in sense of belonging, ultimately crystallizing into pervasive social alienation.

On the basis of the foregoing analysis, the present study posits that among adolescent patients with allergic rhinitis, existential distress first consolidates distressing experiences into rigid cognitive appraisals by heightening psychological inflexibility; subsequently, elevated psychological inflexibility drives individuals to employ experiential avoidance strategies in order to escape unbearable internal experiences; ultimately, experiential avoidance leads to the sustained withdrawal from social interaction contexts, thereby engendering and reinforcing the experience of social alienation. Accordingly, the following hypotheses are proposed:

*H1:* Existential distress is positively associated with social alienation.

*H2:* Psychological inflexibility shows a significant indirect association in the relationship between existential distress and social alienation.

*H3:* Experiential avoidance shows a significant indirect association in the relationship between existential distress and social alienation.

*H4:* Psychological inflexibility and experiential avoidance show a significant serial indirect association between existential distress and social alienation.

## 2 Methods

### 2.1 Study design

This study employed a cross-sectional survey design to examine the relationship between existential distress and social alienation among adolescent patients with allergic rhinitis. The study protocol was reviewed and approved by the Medical Ethics Review Committee of the First Affiliated Hospital of Chengdu Medical College (No. 2025CYFYIRB-BA-156) prior to data collection, and the entire research process was conducted in strict accordance with the Declaration of Helsinki.

Participant recruitment was conducted between December 2025 and February 2026 at the otorhinolaryngology and allergy outpatient departments of three tertiary Grade-A general hospitals in Sichuan Province, China. A convenience sampling method was adopted, whereby research personnel approached eligible adolescent patients and their guardians in the outpatient waiting areas for initial screening. Researchers preliminarily identified adolescent patients aged 12–18 years who presented with allergic rhinitis-related symptoms by consulting the outpatient electronic medical record system. The researchers provided target patients and their guardians with detailed information regarding the study objectives, content, and data confidentiality measures, and furnished written informed consent forms (both patients and guardians were required to provide signatures). Subsequently, patients who met all inclusion criteria and completed the informed consent process were asked to complete a paper-based self-report questionnaire. Research personnel conducted a preliminary completeness check of each questionnaire on site at the time of collection, and questionnaires with obvious missing responses were excluded.

The inclusion criteria were as follows: (1) aged between 12 and 18 years (inclusive); (2) diagnosed with allergic rhinitis by an otorhinolaryngology specialist; (3) allergic rhinitis duration of ≥5 months, which was used as a study-specific operational criterion to ensure a relatively sustained illness experience; (4) adequate Chinese literacy to independently comprehend and complete the study questionnaire; and (5) written informed consent obtained from both the patient and their legal guardian.

The criterion requiring an allergic rhinitis duration of ≥5 months was adopted as an operational eligibility criterion rather than as a substitute for the ARIA classification of persistent allergic rhinitis. In the present study, the diagnosis of allergic rhinitis was established by otorhinolaryngology specialists according to patients’ clinical manifestations and medical records, whereas the duration criterion was used to ensure that participants had experienced a relatively sustained and recurrent illness course sufficient for the development of stable psychosocial responses. This cutoff was determined before formal data collection on the basis of clinical discussions with otorhinolaryngology specialists and a preliminary feasibility assessment conducted in the outpatient setting. During the preparatory phase, adolescents with very recent onset of allergic rhinitis tended to describe mainly transient physical discomfort and short-term emotional reactions, whereas those with several months of recurrent symptoms more frequently reported illness-related restrictions in daily activities, concerns about uncontrollability, and difficulties in social participation. Therefore, a duration of ≥5 months was selected to reduce the likelihood of including newly diagnosed or transient cases and to increase the homogeneity of participants with respect to chronic illness experience. Nevertheless, this threshold should be interpreted as a study-specific operational definition rather than a universally established clinical or psychosocial cutoff.

The exclusion criteria were as follows: (1) comorbidity with other severe chronic somatic diseases; (2) a past or current diagnosis of schizophrenia, major depressive disorder, post-traumatic stress disorder, or other severe psychiatric disorders; (3) receipt of systematic psychotherapy or psychological counseling within the preceding 3 months; and (4) evidence of patterned responding or excessive response consistency in the questionnaire.

The minimum required sample size was estimated using G*Power 3.1 software (36). An F-test was employed, with the statistical test specified as “Linear multiple regression: Fixed model, *R*^2^ deviation from zero.” Parameters were set at a medium effect size of *f*^2^ = 0.15, a significance level of *α* = 0.05, a statistical power of 1 − *β* = 0.95, and a maximum of six predictors in the model, yielding a minimum required sample size of 146 participants.

A total of 508 participants were recruited during the data collection period. Of these, 28 participants had an allergic rhinitis duration of less than 5 months, and 19 participants did not personally sign the informed consent form. During the data cleaning process, an additional 36 questionnaires were excluded due to patterned responding. Consequently, the final valid sample comprised 425 participants, yielding an effective response rate of 83.66%. Among these, 258 were male (60.70%) and 167 were female (39.30%). Detailed sociodemographic information is presented in Table 1.

**Table 1:** Sociodemographic characteristics of adolescent patients with allergic rhinitis.

Variables	Items	Number (N)	Proportion (%)
Gender	Male	258	60.7%
	Female	167	39.3%
Educational level	Primary school and below	77	18.1%
	Junior high school	189	44.5%
	High school	159	37.4%
Place of residence	Urban	252	59.3%
	Rural area	173	40.7%
Only child	No	270	63.5%
	Yes	155	36.5%
Disease duration	0.5–1 year	90	21.2%
	1–3 year	165	38.8%
	3 year and above	170	40.0%
Age		15.59 ± 1.931	

Basic sociodemographic information and disease duration were collected using a self-designed questionnaire. However, standardized measures of allergic rhinitis severity, symptom control, and treatment status, including symptom burden, current medication use, immunotherapy, and treatment adherence, were not systematically assessed.

### 2.2 Measurement instruments

#### 2.2.1 Existential distress scale

The Chinese version of the Existential Distress Scale was adapted by Huimin et al. (37) and comprises 10 items encompassing three dimensions: loneliness, meaninglessness, and worthlessness. A sample item is “Allergic rhinitis makes me feel lonely.” This scale was used in the present study to assess existential distress among adolescent patients with allergic rhinitis. Responses were rated on a 5-point Likert scale ranging from 1 (“no distress”) to 5 (“unbearable distress”). Total scores range from 10 to 50, with higher scores indicating greater levels of existential distress. In the present study, the Cronbach’s alpha coefficient for this scale was 0.837, indicating good internal consistency. Confirmatory factor analysis found that the model of existential distress had an excellent fit (*χ*^2^/df = 1.832, GFI = 0.971, AGFI = 0.955, RMSEA = 0.044, CFI = 0.972, TLI = 0.964).

#### 2.2.2 Social alienation scale

The Social Alienation Scale was originally developed Jessor and Jessor (38) and was translated into Chinese by Wu et al. (39). The scale consists of 15 items spanning four dimensions: alienation from others, suspiciousness, self-alienation, and meaninglessness. A sample item is “I sometimes feel that the people I know are not very friendly.” This scale was employed in the present study to measure social alienation among adolescent patients with allergic rhinitis. Responses were rated on a 5-point Likert scale ranging from 1 (“strongly disagree”) to 5 (“strongly agree”). Total scores range from 15 to 75, with higher scores reflecting greater levels of social alienation. In the present study, the Cronbach’s alpha coefficient for this scale was 0.871, indicating good internal consistency. Confirmatory factor analysis found that the model of social alienation had an excellent fit (*χ*^2^/df = 1.013, GFI = 0.972, AGFI = 0.962, RMSEA = 0.016, CFI = 0.989, TLI = 0.989).

#### 2.2.3 Psychological inflexibility scale

The Psychological Inflexibility Scale was originally developed by Greco et al. (40) and was translated into Chinese by Chen et al. (41). The scale comprises 8 items and is unidimensional. A sample item is “My life won’t be good until I feel happy.” This scale was used in the present study to assess psychological inflexibility among adolescent patients with allergic rhinitis. Responses were rated on a 5-point Likert scale ranging from 1 (“not at all like me”) to 5 (“exactly like me”). Total scores range from 8 to 40, with higher scores indicating greater psychological inflexibility and lower psychological flexibility. In the present study, the Cronbach’s alpha coefficient for this scale was 0.843, indicating good internal consistency. Confirmatory factor analysis found that the model of psychological inflexibility had an excellent fit (*χ*^2^/df = 1.161, GFI = 0.987, AGFI = 0.976, RMSEA = 0.019, CFI = 0.995, TLI = 0.993).

#### 2.2.4 Experiential avoidance scale

The Experiential Avoidance Scale was originally developed by Gámez et al. (42) and was translated into Chinese by Cao et al. (43). The scale consists of 15 items encompassing two dimensions: cognitive avoidance and behavioral avoidance. A sample item is “I quickly move away from situations that make me feel nervous or uneasy.” This scale was used in the present study to assess experiential avoidance among adolescent patients with allergic rhinitis. Responses were rated on a 5-point Likert scale ranging from 1 (“strongly disagree”) to 5 (“strongly agree”). Total scores range from 15 to 75, with higher scores indicating more severe experiential avoidance. In the present study, the Cronbach’s alpha coefficient for this scale was 0.858, indicating good internal consistency. Confirmatory factor analysis found that the model of experiential avoidance had an excellent fit (*χ*^2^/df = 1.063, GFI = 0.971, AGFI = 0.962, RMSEA = 0.012, CFI = 0.986, TLI = 0.985).

### 2.3 Statistical analysis

All statistical analyses were performed using SPSS 27.0 software, and the serial mediation analysis was conducted using the PROCESS macro (version 4.1). First, Harman’s single-factor test was employed to examine common method bias. Subsequently, descriptive statistics and correlation analyses were conducted for the core study variables. Third, PROCESS Model 6 was applied to examine the hypothesized serial indirect associations among existential distress, psychological inflexibility, experiential avoidance, and social alienation. Because all variables were measured at the same time point, the mediation analysis was interpreted as a cross-sectional statistical model and was not used to infer temporal precedence or causality. The bias-corrected percentile bootstrap method with 5,000 resamples was used to estimate 95% confidence intervals for all path coefficients.

## 3 Results

### 3.1 Common method bias test

Given that self-report methods may be susceptible to common method bias, several procedural remedies were implemented during the research design phase. Specifically, anonymous administration was adopted to reduce social desirability bias, the measurement instruments for each variable were randomly ordered within the questionnaire to minimize order effects, and the instructions explicitly informed participants that there were no right or wrong answers to encourage truthful responding.

In addition, Harman’s single-factor test was conducted by entering all measurement items into an unrotated exploratory factor analysis. The results revealed eight factors with eigenvalues greater than 1, and the first factor accounted for 22.881% of the total variance, which was well below the critical threshold of 40%. Therefore, common method bias was unlikely to pose a serious threat to the present study data.

### 3.2 Descriptive statistics and correlation analysis

The descriptive statistics and correlation analysis results for the core study variables are presented in Table 2. Skewness values for the core study variables ranged from −0.177 to −0.096, and kurtosis values ranged from −0.608 to −0.537, satisfying the criteria for approximate normality (skewness ≤ |3|, kurtosis ≤ |8|) (44). The correlation analysis revealed that existential distress was significantly and positively correlated with social alienation (*r* = 0.479, *p* < 0.001), psychological inflexibility (*r* = 0.509, *p* < 0.001), and experiential avoidance (*r* = 0.532, *p* < 0.001). Psychological inflexibility was significantly and positively correlated with experiential avoidance (*r* = 0.424, *p* < 0.001) and social alienation (*r* = 0.533, *p* < 0.001). Experiential avoidance was significantly and positively correlated with social alienation (*r* = 0.434, *p* < 0.001).

**Table 2:** Descriptive statistics and correlation analysis results for core study variables.

Variables	*M*	SD	Skewness	Kurtosis	1	2	3	4
1. Existential distress	3.340	0.789	−0.177	−0.537	1			
2. Social alienation	3.228	0.734	−0.096	−0.549	0.479***	1		
3. Psychological inflexibility	3.304	0.772	−0.132	−0.545	0.509***	0.533***	1	
4. Experiential avoidance	3.281	0.727	−0.149	−0.608	0.532***	0.434***	0.424***	1

***p < 0.001; M = Mean; SD = Standard Deviation.

### 3.3 Cross-section serial mediation analysis of psychological inflexibility and experiential avoidance

**Figure 1:**
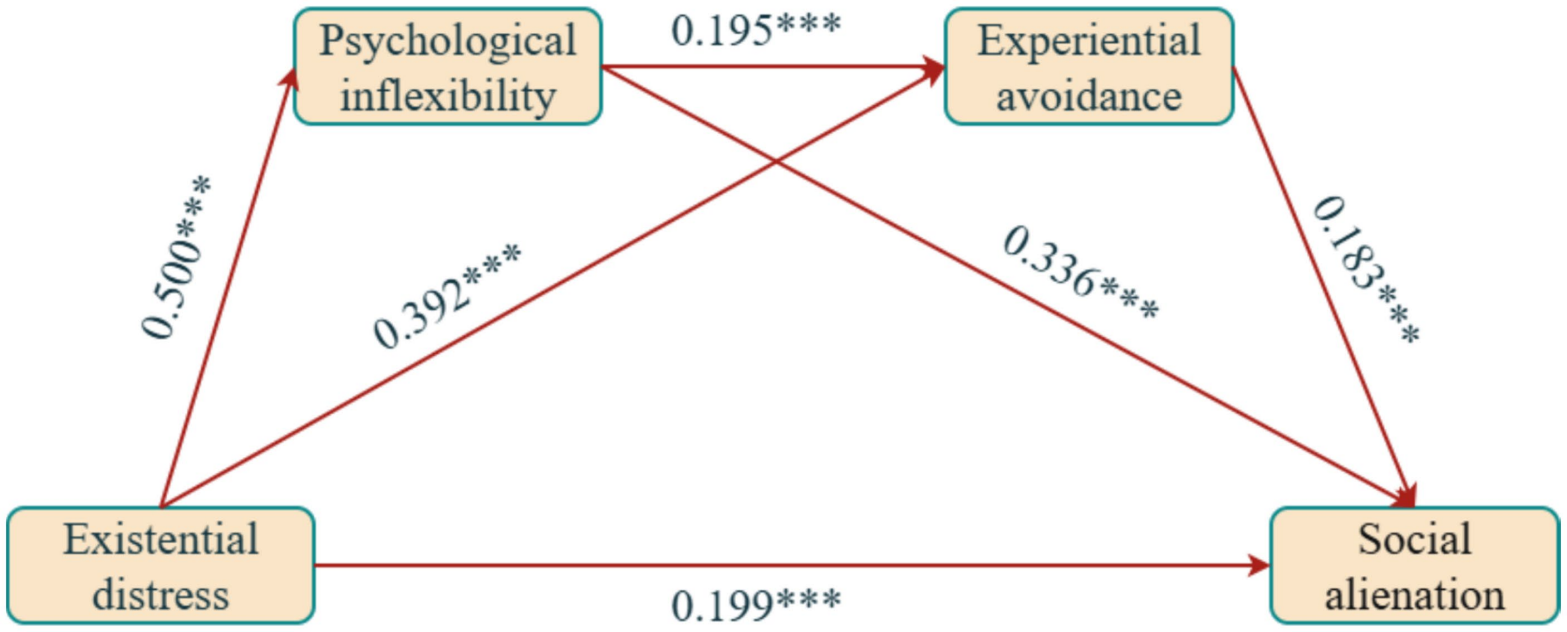
Path coefficients of the serial mediation model of psychological inflexibility and experiential avoidance. ***p < 0.001.

To examine the cross-section serial mediating effects of psychological inflexibility and experiential avoidance in the relationship between existential distress and social alienation, PROCESS Model 6 was employed. Age, educational level, and disease duration were controlled as covariates. Three regression equations were sequentially constructed using hierarchical regression, and the significance of each indirect effect was tested using the bias-corrected percentile bootstrap method with 5,000 resamples. The specific results are presented in Table 3 and Figure 1.

**Table 3:** Regression analysis of psychological inflexibility and experiential avoidance.

Model	Outcome	Predictor	*B*	SE	*p*	LLCI	ULCI	*R* ^2^	*F*
Model 1	Psychological inflexibility	Existential distress	0.500	0.042	<0.001	0.418	0.583	0.262	29.755
		Age	0.007	0.017	0.694	−0.027	0.040		
		Education level	−0.028	0.043	0.515	−0.114	0.057		
		Disease duration	−0.050	0.043	0.247	−0.134	0.035		
Model 2	Experiential avoidance	Existential distress	0.392	0.044	<0.001	0.305	0.478	0.319	32.641
		Psychological inflexibility	0.195	0.044	<0.001	0.108	0.282		
		Age	−0.007	0.015	0.645	−0.037	0.023		
		Education level	−0.051	0.039	0.200	−0.128	0.027		
		Disease duration	0.030	0.039	0.444	−0.047	0.106		
Model 3	Social alienation	Existential distress	0.199	0.046	<0.001	0.108	0.290	0.379	36.329
		Psychological inflexibility	0.336	0.044	<0.001	0.250	0.422		
		Experiential avoidance	0.183	0.047	<0.001	0.090	0.276		
		Age	−0.029	0.015	0.051	−0.058	0.000		
		Education level	0.080	0.038	0.037	0.005	0.155		
		Disease duration	−0.059	0.038	0.118	−0.133	0.015		

Psychological inflexibility. After controlling for demographic and clinical covariates, the overall model was statistically significant (*R*^2^ = 0.262, *F* = 29.755), indicating that the combination of predictors in this equation explained 26.2% of the total variance in psychological inflexibility. Existential distress was significantly and positively associated with psychological inflexibility (*B* = 0.500, *p* < 0.001, 95% CI [0.418, 0.583]).

Experiential avoidance. After controlling for covariates, the overall model was statistically significant (*R*^2^ = 0.319, *F* = 32.641), with the combination of predictors explaining 31.9% of the total variance in experiential avoidance. The predictive effect of existential distress on experiential avoidance was significant (*B* = 0.392, *p* < 0.001, 95% CI [0.305, 0.478]), indicating that after controlling for the mediating role of psychological inflexibility, existential distress remained significantly and positively associated with experiential avoidance. Concurrently, psychological inflexibility also exerted a significant positive predictive effect on experiential avoidance (*B* = 0.195, *p* < 0.001, 95% CI [0.108, 0.282]), confirming that elevated levels of psychological inflexibility can further exacerbate experiential avoidance.

Social alienation. Upon simultaneously entering the independent variable and both mediating variables, the explanatory power of the overall model was further enhanced (*R*^2^ = 0.379, *F* = 36.329), with the combination of predictors explaining 37.9% of the total variance in social alienation. The direct association between existential distress on social alienation remained significant (*B* = 0.199, *p* < 0.001, 95% CI [0.108, 0.290]). Psychological inflexibility was significantly and positively associated with social alienation (*B* = 0.336, *p* < 0.001, 95% CI [0.250, 0.422]). Experiential avoidance was also significantly and positively associated with social alienation (*B* = 0.183, *p* < 0.001, 95% CI [0.090, 0.276]).

The bias-corrected percentile bootstrap method (5,000 resamples) was further employed to test the three indirect effect pathways and the total indirect effect, with the results summarized in Table 4. The total effect of existential distress on social alienation was 0.457, of which the direct effect of the “existential distress → social alienation” pathway accounted for 43.5%, and the total indirect effect accounted for 56.5%. Specifically, the pathway “existential distress → psychological inflexibility → social alienation” accounted for 36.9% of the total effect, indicating that psychological inflexibility served as a significant mediator between existential distress and social alienation (Effect = 0.168, SE = 0.028, 95% CI [0.116, 0.226]). The pathway “existential distress → experiential avoidance → social alienation” accounted for 15.7% of the total effect, indicating that experiential avoidance served as a significant mediator between existential distress and social alienation (Effect = 0.072, SE = 0.022, 95% CI [0.031, 0.119]). The pathway “existential distress → psychological inflexibility → experiential avoidance → social alienation” accounted for 3.9% of the total effect, indicating that psychological inflexibility and experiential avoidance jointly served as significant serial mediators between existential distress and social alienation.

**Table 4:** Decomposition of direct and indirect associations in the cross-sectional serial mediation model.

Type of effect	Effect	SE	LLCI	ULCI	Proportion of effect	Supporting hypothesis
Total effect	0.457	0.040	0.377	0.536	100%	
Direct effect	0.199	0.046	0.108	0.290	43.5%	H1
Total indirect effect	0.258	0.036	0.188	0.333	56.5%	
Ind1	0.168	0.028	0.116	0.226	36.9%	H2
Ind2	0.072	0.022	0.031	0.119	15.7%	H3
Ind3	0.018	0.007	0.006	0.033	3.9%	H4

Ind 1 = Existential distress → Psychological inflexibility → Social alienation; Ind 2 = Existential distress → Experiential avoidance → Social alienation; Ind 3 = Existential distress → Psychological inflexibility → Experiential avoidance → Social alienation.

## 4 Discussion

### 4.1 Theoretical implications

The present study revealed a significant positive association between existential distress and social alienation among adolescent patients with allergic rhinitis. Previous research on existential distress has predominantly focused on severe somatic conditions such as cancer and terminal illnesses (45, 46). However, the findings of this study demonstrate that adolescent allergic rhinitis patients also report clinically meaningful levels of existential distress, and that this distress experience bears a substantive association with their social functioning. This suggests that the emergence of existential distress is not solely contingent upon the severity or lethality of a disease (47), but rather depends more critically on the extent to which the illness experience triggers an individual’s core existential concerns.

The present study supported a significant cross-sectional indirect association involving psychological inflexibility in the relationship between existential distress and social alienation. Specifically, existential distress among adolescent allergic rhinitis patients intensifies profound questioning regarding self-worth and the meaning of life (48). Under conditions of high psychological inflexibility, such questioning is no longer perceived and accepted by the individual as a transient psychological experience; instead, through the process of cognitive fusion, it is experienced as immutable truths about the self and the world. When an individual’s behavior is no longer guided by personal values and contextual flexibility but is instead dominated by these rigid internal narratives, the individual becomes more inclined toward withdrawal and isolation in social interactions, thereby experiencing elevated levels of social alienation.

The present study also supported a significant cross-sectional indirect association involving experiential avoidance in the relationship between existential distress and social alienation. According to Acceptance and Commitment Therapy theory, experiential avoidance is a functionally oriented contextual control strategy whereby individuals avoid situations that elicit internal distress to achieve short-term emotional relief (49), at the cost of progressive impairment in long-term social functioning. In the context of adolescent allergic rhinitis patients, the internal discomfort elicited by existential distress may directly drive individuals to adopt experiential avoidance strategies (50). For example, patients may proactively avoid social situations that are likely to activate existential distress. Although avoidance strategies may effectively reduce the subjective intensity of existential distress in the short term, their long-term functional consequence is a systematic erosion of opportunities for social interaction. Over time, diminished social participation leads to weakened social bonds, deterioration of social skills, and declining quality of peer relationships, ultimately becoming internalized as a pervasive experience of social alienation.

It is worth further discussing that the mediating effect of experiential avoidance was smaller than that of psychological inflexibility. This finding provides empirical evidence suggesting that, in the process through which existential distress is transformed into social alienation, cognitive-level rigidity processes may exert a stronger transmitting effect than behavioral-level avoidance strategies. This may be attributable to the fact that psychological inflexibility, as a more fundamental and pervasive psychological process, extends its influence beyond specific behavioral contexts and permeates the individual’s overarching self-concept, value orientation, and life narrative, thereby reshaping social cognition and interaction patterns at a deeper level. By contrast, although experiential avoidance similarly results in social functioning impairment, its sphere of influence is more confined to specific behavioral and situational domains.

### 4.2 Practical implications

The empirical findings of the present study provide multi-layered practical insights for the clinical assessment and intervention of social alienation among adolescent allergic rhinitis patients. First, the results suggest that clinical practitioners should incorporate systematic screening for existential distress into routine assessments for adolescent allergic rhinitis patients. This study found that adolescent allergic rhinitis patients reported clinically meaningful levels of existential distress, and that this distress experience significantly predicted their social alienation through multiple pathways. For clinicians engaged in adolescent mental health services, even when dealing with seemingly mild chronic conditions such as allergic rhinitis, vigilance should be maintained regarding the potential presence of deep-seated psychological distress concerning self-worth, life meaning, and existential fairness among adolescent patients. In terms of assessment strategy, clinicians should incorporate structured screening of the existential distress dimension into routine psychological assessments to facilitate early identification and intervention for adolescent patients at high risk for existential distress.

Second, the finding regarding the central mediating role of psychological inflexibility provides clear target-oriented guidance for the design of clinical intervention strategies. Given limited clinical resources and intervention time, prioritizing interventions targeting psychological inflexibility may yield the greatest clinical benefit. Specifically, cognitive defusion techniques grounded in Acceptance and Commitment Therapy may represent the most effective intervention entry point for reducing social alienation among adolescent allergic rhinitis patients. Unlike traditional cognitive restructuring strategies, cognitive defusion does not attempt to alter the content or frequency of existential thoughts; rather, it modifies the relational pattern between the individual and these thoughts, enabling the individual to maintain behavioral flexibility and value-directed action while still experiencing existential distress.

The chain mediating pathway involving psychological inflexibility and experiential avoidance offers process-oriented guidance for the sequential design of intervention programs. This suggests that the design of intervention programs should follow a logical sequence of cognitive defusion first, followed by behavioral activation. During the initial phase of intervention, clinicians should focus on employing cognitive defusion and mindful acceptance techniques from Acceptance and Commitment Therapy to help adolescent patients reduce their levels of psychological inflexibility and establish a flexibly accepting relationship with their existential distress experiences. Building upon this foundation, the intervention program can further introduce value-directed behavioral activation strategies, guiding patients to proactively engage in social activities in accordance with their personal values while maintaining awareness and acceptance of internal distress experiences.

### 4.3 Limitations and future research directions

Despite the aforementioned theoretical and practical contributions, the present study is subject to several limitations. First, this study employed a cross-sectional survey design in which all study variables were measured simultaneously at a single time point, which inherently constrains inferences regarding the causal direction of the relationships among variables. For instance, social alienation may in turn exacerbate existential distress experiences, forming a positive feedback loop. Future research should adopt longitudinal tracking designs or cross-lagged panel designs, measuring each study variable at multiple time points, to more rigorously examine the temporal relationships and causal directions among variables.

Second, the data in this study were derived entirely from participant self-reports and may be susceptible to common method bias. Although multiple procedural control measures were implemented during the research design phase and the results of Harman’s single-factor test indicated that common method bias was not a serious concern, the inherent limitations of self-report methodology may still affect the accuracy of the findings to some extent. Future research may consider adopting multi-method measurement strategies, such as integrating behavioral experimental tasks (e.g., the Implicit Association Test for assessing the degree of cognitive fusion), ecological momentary assessment, and informant reports (e.g., parent or teacher evaluations of changes in social behavior), to enhance ecological validity and construct validity.

Third, the participant sample in this study was recruited through convenience sampling from the outpatient departments of three tertiary general hospitals in Sichuan Province, China, which limits the external generalizability of the research conclusions to some degree. Future research should conduct cross-sample validation across broader geographical regions and diverse cultural contexts and include community-based samples to improve sample representativeness.

Fourth, although the mediation model in this study encompassed two core mediating variables—psychological inflexibility and experiential avoidance—from a theoretical standpoint, psychosocial factors such as identity crises, illness-related self-stigmatization, emotion regulation difficulties, ruminative thinking, and perceived social support may all potentially serve as mediating or moderating roles in the relationship between existential distress and social alienation. Future research could construct more comprehensive multiple mediation or moderated mediation models incorporating these potential variables to more fully elucidate the psychological mechanism network through which existential distress is transformed into social alienation.

Fifth, the duration criterion of ≥5 months should be interpreted with caution. Although this criterion was used to ensure that participants had experienced a relatively sustained and recurrent course of allergic rhinitis and was determined before data collection on the basis of clinical discussion and feasibility considerations, it is not an ARIA-defined threshold and has not been validated as a clinically meaningful cutoff for the emergence of existential distress or social alienation. Therefore, the present findings may not be directly generalizable to adolescents with newly diagnosed allergic rhinitis or a shorter duration of symptoms. Future studies should treat disease duration as a continuous variable, compare different duration-based subgroups, and incorporate ARIA-based classifications of intermittent and persistent allergic rhinitis to clarify whether and how illness duration and symptom persistence contribute to existential distress and social alienation over time.

Although allergic rhinitis is a chronic condition with fluctuating symptom burden, the present study did not systematically assess disease severity, symptom control, or detailed treatment status. These clinical variables may influence adolescents’ psychological distress, avoidance tendencies, and social functioning. For example, adolescents with more severe or poorly controlled symptoms may experience greater sleep disturbance, fatigue, embarrassment, academic impairment, and peer interaction difficulties, which may in turn be associated with higher existential distress and social alienation. Similarly, treatment status, medication adherence, and response to therapy may affect symptom burden and psychological adjustment. Therefore, the absence of these clinical indicators limits our ability to determine whether the observed associations remain stable across different levels of allergic rhinitis severity or treatment control. Future studies should incorporate standardized clinical assessments, such as ARIA-based severity classification, symptom control measures, medication use, immunotherapy status, and treatment adherence, to clarify the role of disease-related factors in psychological and social outcomes among adolescents with allergic rhinitis.

## 5 Conclusion

The present study examined the association between existential distress and social alienation and explored theoretically proposed psychosocial processes linking these variables among adolescent patients with allergic rhinitis. The results showed that existential distress was significantly and positively associated with social alienation. In a cross-sectional serial mediation model, psychological inflexibility and experiential avoidance were identified as significant statistical mediators in this association. These findings provide preliminary support for extending existential distress research from severe somatic illnesses to non-life-threatening chronic disease populations and suggest that ACT-related processes may be relevant to social functioning difficulties in adolescents with allergic rhinitis. Future longitudinal and intervention studies are needed to determine whether reducing psychological inflexibility and experiential avoidance can alleviate social alienation in this population.

## Data Availability

The raw data supporting the conclusions of this article will be made available by the authors, without undue reservation.
